# Supplementary material for: Working memory signals in early visual cortex are present in weak and strong imagers
Source: Hum Brain Mapp. 2024 Feb 24;45(3):e26590. doi: 10.1002/hbm.26590 (PMC10893972; doi:10.1002/hbm.26590)
Supplement: Supplementary file 1 — Data S1. Supporting Information. [file HBM-45-e26590-s001.pdf]

Supplemental Information

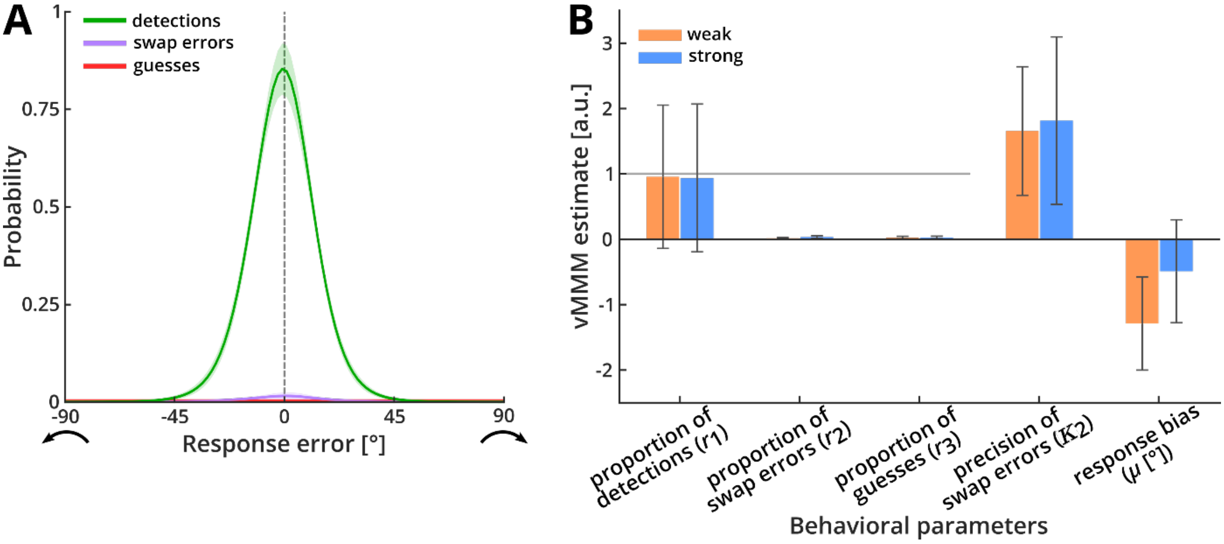

Figure S1. Von Mises mixture model (vMMM) fit of behavioral responses. (A) The distribution of behavioral responses was modeled as a combination of the three model components: detections (responses to target orientations, assumed to follow a von Mises distribution with mean 0° plus bias μ and precision κ; green), swap errors (responses to distractor orientations, following the same assumptions as detections; purple) and guesses (assumed to follow a continuous uniform distribution between -90° and +90°; red). These components were weighted by individual event probabilities (mixture coefficients) r_1, r_2 and r_3, respectively. Participants correctly responded to the target direction in 94.7 % of trials (r_1 = 0.947 ± 0.063), and only infrequently made swap errors (r_2 = 0.026 ± 0.034) or guesses (r_3 = 0.027 ± 0.041). Responses to targets were precise (κ_1 = 5.673 ± 2.377), while responses to the distractor, where present, were imprecise (κ_2 = 1.735 ± 2.41). There was a small but significant bias to respond anti-clockwise of the target (μ = -0.889 ± 1.635°; $t_{(39)}$ = -3.437, p = 0.0014, two-tailed; see also Figure 1C). **(B)** Estimated vMMM parameters for strong and weak imagers separately. There was no significant difference between the two groups for any of the estimated parameters (r_1: $t_{(38)}$ = -0.925, p = 0.361; r_2: $t_{(38)}$ = 1.585, p = 0.121; r_3: $t_{(38)}$ = 0.108, p = 0.914; $\kappa_{2:}$ $t_{(38)}$ = -0.207, p = 0.837; μ: $t_{(38)}$ = 1.574, p = 0.124, all two-tailed; see Figure 1D for κ_1).

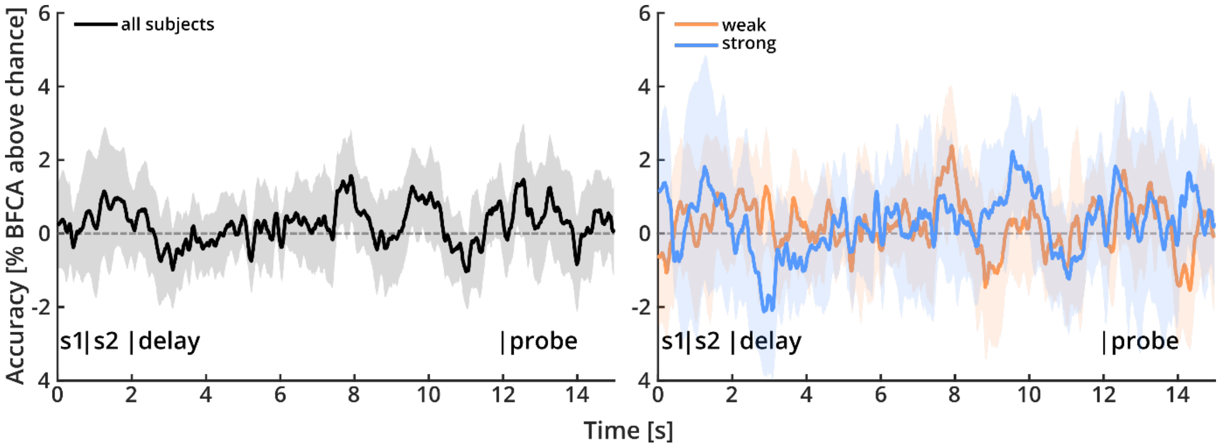

Figure S2. Target reconstruction from eye-tracking data. Reconstruction of target orientation from gaze position across the trial, for all subjects (left panel) and separated by groups (right panel). There were no temporal clusters with significantly above-chance BFCA, suggesting that participants did not systematically use gaze position to maintain target orientation across the delay period. Shaded areas indicate 95 % confidence intervals.

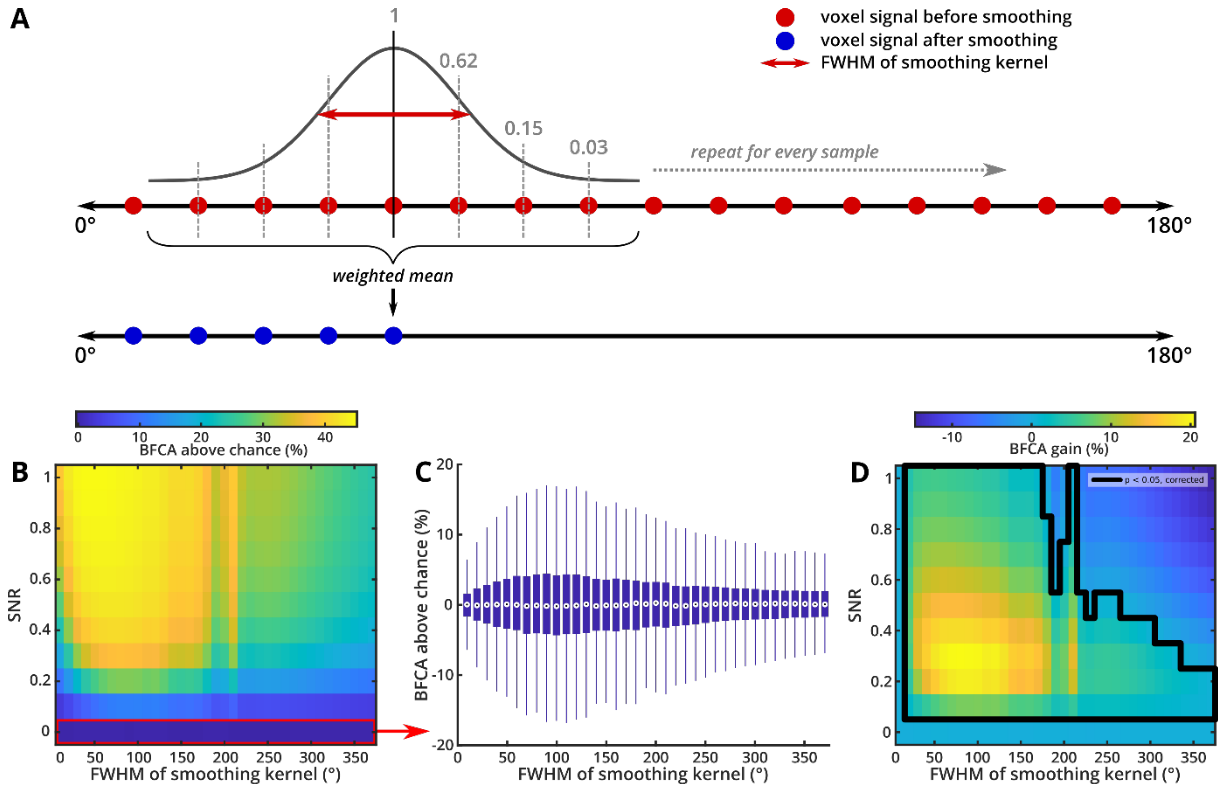

Figure S3. Schematic representation of feature-space smoothing and simulation results. (A) We used a Gaussian smoothing kernel to compute a weighted average from the voxel signal of samples lying closely together in feature space. Samples close to a given orientation in feature-space therefore contribute more to the resulting average than those further away. The full width at half maximum (FWHM) of the smoothing kernel controls the smoothing range, i.e., the number (or distance) of samples that are included in the weighted average. We used FWHM values between 0° (no smoothing) and 90° in steps of 10° and determined the optimal kernel width for each participant via nested cross-validation across subjects. Note that this was done (a) at the level of the input data to the analysis, not the results, (b) for training and test data separately, and (c) was confirmed not to produce artifacts or spurious results by extensive simulations (see (C) and Extended Methods). **(B)** We simulated data with varying levels of SNR and used feature-space smoothing with different kernel widths (measured as FWHM in degrees) before reconstruction of the underlying signal. The plot shows BFCA for all parameter combinations, averaged across 1000 repetitions. **(C)** BFCA across smoothing levels, for the pure noise condition. BFCA remained at chance-level across all levels of smoothing (all $p > 0.25$) and BFCA for any smoothing condition did not differ from the no-smoothing condition (all $p > 0.15$). **(D)** BFCA gain compared to no smoothing, averaged across all 1000 repetitions. The first column corresponds to baseline, i.e., zero smoothing. In the signal conditions ($\mathrm{SNR} > 0$), feature-space smoothing was able to reliably increase BFCA compared to no smoothing. The effect was strongest for smoothing kernel widths between 30° and 170°, where we observed increases in accuracy of up to 20%. Generally, the effect of feature space smoothing was stronger for data with low SNR (orange-yellow area). In cases of extremely high kernel-width and comparatively high SNR (i.e., $\mathrm{SNR} > 0.6$ and $\mathrm{FWHM} > 220°$), feature-space smoothing had a detrimental effect, meaning that BFCA was decreased compared to no smoothing (dark blue area). Please note, however, that kernel-widths this high do not make any sense for real-world applications and were only included for the purpose of demonstration. We conclude that feature-space smoothing is a powerful preprocessing technique to increase SNR in a feature-continuous reconstruction setting. As the optimal kernel-width for smoothing depends on the specific data and SNR, we recommend using nested cross-validation to determine the optimal FWHM value, similar to the approach described in the main text.

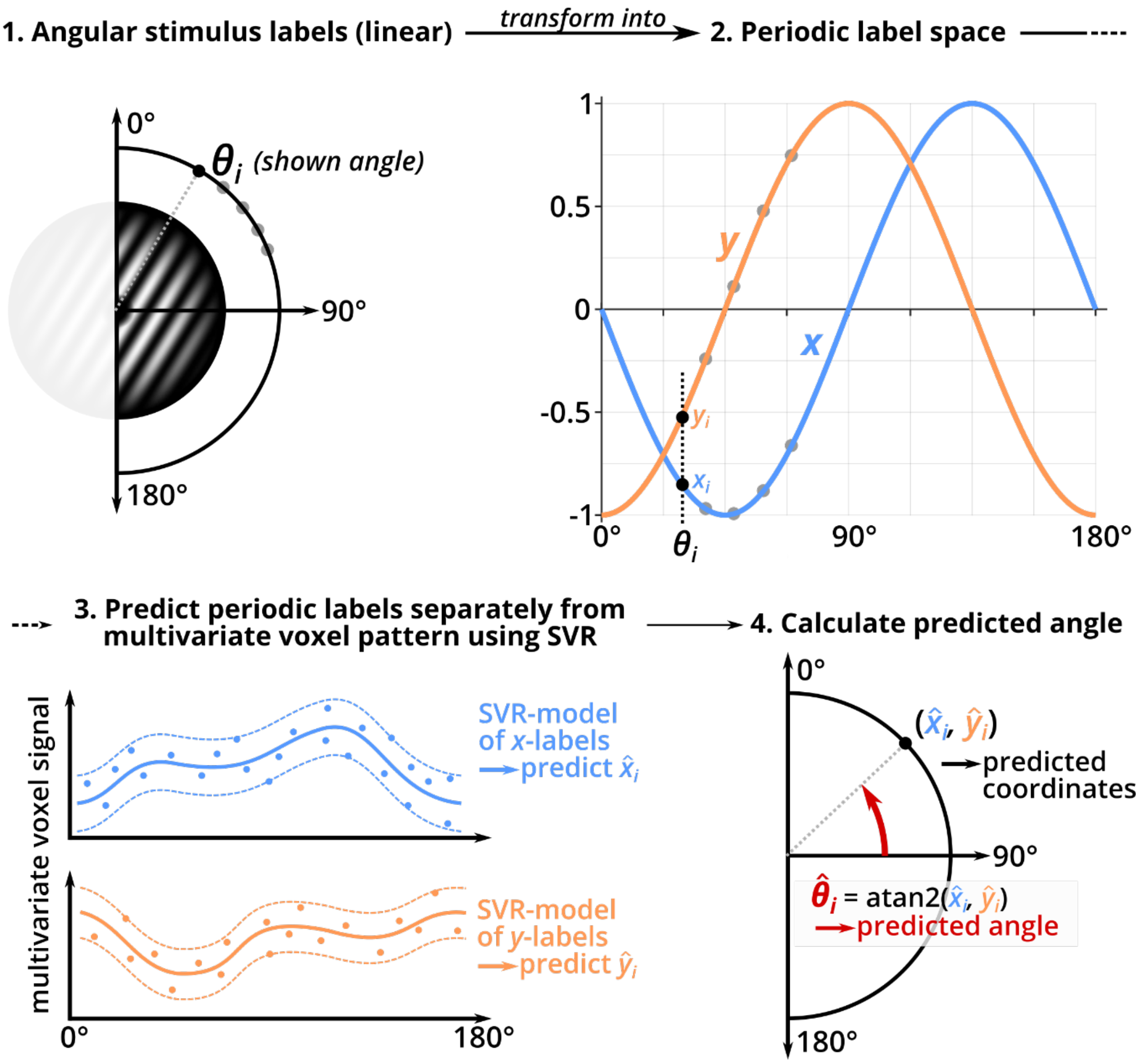

Figure S4. Schematic representation of periodic support vector regression (pSVR). The aim of our reconstruction analysis was to predict an angular label between 0° and 180° from the multivariate voxel signal in response to a stimulus grating with the respective orientation. However, the linear scale of orientation labels (from 0° to 180°) does not reflect the periodic nature of the stimulus (i.e., 0° and 180° are identical). To account for this, we projected the angular labels into a periodic space by fitting two sinusoids into the range [0, 180). Both functions had an amplitude of 1 and a period of 180°, so that one period spanned the entire label space. One function was shifted by 45°, so that the combination of both periodic functions coded for the linear label scale. This is equivalent to the way sine and cosine functions between 0 and 360° code for the angles on a unit circle. We trained and tested a multivariate SVR model for both periodic label sets (x, y) separately. From the combination of the predicted periodic labels, we then reconstructed a predicted angular label using the four-quadrant inverse tangent. The predicted orientation was then compared to the true orientation to derive BFCA, our measure of reconstruction accuracy.

Table S1: Correlation table of all variables of interest (and strategy questionnaire).

	BFCA target	Behav. precision	Pre-scan VVIQ	Post-scan VVIQ	OSIQ visual	OSIQ spatial	Strat. visual	Strat. verbal	Strat. spatial	Strat. cardinal	Strat. clock	Strat. code	Strat. other
**BFCA target**		0.728***	-0.256	-0.277	-0.297	0.166	-0.203	0.245	-0.289	-0.146	0.173	-0.174	0.402*
**Behav. precision**	0.728***		-0.127	-0.062	-0.158	-0.011	-0.118	0.274	-0.179	-0.268	0.044	0.061	0.235
**Pre-scan VVIQ**	-0.256	-0.127		0.867***	0.706***	-0.271	0.109	-0.182	0.073	0.293	0.06	-0.011	-0.43**
**Post-scan VVIQ**	-0.277	-0.062	0.867***		0.837***	-0.25	0.157	-0.208	0.005	0.246	0.114	0.029	-0.488**
**OSIQ visual**	-0.297	-0.158	0.706***	0.837***		-0.239	0.273	-0.319*	0.004	0.241	0.089	0.067	-0.564***
**OSIQ spatial**	0.166	-0.011	-0.271	-0.25	-0.239		-0.011	0.006	0.125	0.128	-0.238	-0.21	0.28
**Strat. visual**	-0.203	-0.118	0.109	0.157	0.273	-0.011		-0.427**	-0.271	-0.371*	-0.119	-0.233	-0.365*
**Strat. verbal**	0.245	0.274	-0.182	-0.208	-0.319*	0.006	-0.427**		-0.009	-0.023	-0.316*	0.064	0.14
**Strat. spatial**	-0.289	-0.179	0.073	0.005	0.004	0.125	-0.271	-0.009		0.195	-0.436**	0.032	-0.051
**Strat. cardinal**	-0.146	-0.268	0.293	0.246	0.241	0.128	-0.371*	-0.023	0.195		-0.247	-0.01	-0.077
**Strat. clock**	0.173	0.044	0.06	0.114	0.089	-0.238	-0.119	-0.316*	-0.436**	-0.247		-0.15	-0.144
**Strat. code**	-0.174	0.061	-0.011	0.029	0.067	-0.21	-0.233	0.064	0.032	-0.01	-0.15		-0.21
**Strat. other**	0.402*	0.235	-0.43**	-0.488**	-0.564***	0.28	-0.365*	0.14	-0.051	-0.077	-0.144	-0.21	

Table of correlation coefficients between all variables of interest, including the items from the heuristic strategy questionnaire. There are two notable sets of relationships: the strong correlation between target reconstruction accuracy (“BFCA target”) and behavioral precision (“Behav. precision”), and the close relationship between pre- and post-scan VVIQ (i.e., test-retest reliability) and the visual OSIQ scores. There are some significant effects between several variables and items from the strategy (“Strat.”) questionnaire. Please note, however, that these questions were purely heuristic in nature. We only asked for each strategy in rather general terms and did not ask for the vividness of each strategy. The questions were not based on any previously validated procedure, in contrast to the established VVIQ and OSIQ scales. Also, the ratings on these items have high variance, rendering any interpretation difficult. We are currently not aware of any established and standardized sets of questions regarding the use of cognitive strategies.

Table S2: Descriptive statistics for all variables of interest (and strategy questionnaire).

	BFCA target	Behav. precision	Pre-scan VVIQ	Post-scan VVIQ	OSIQ visual	OSIQ spatial	Strat. visual	Strat. verbal	Strat. spatial	Strat. cardinal	Strat. clock	Strat. code	Strat. other
**Mean**	12.21	5.673	55.725	58.25	49.525	44.225	0.285	0.141	0.189	0.071	0.186	0.054	0.073
**Standard deviation**	9.768	2.377	17.332	15.834	12.878	8.636	0.217	0.13	0.119	0.115	0.179	0.092	0.121
**Skewness**	0.484	1.143	-0.641	-1.055	-1.048	-0.187	1.664	0.453	0.062	1.758	0.435	1.335	1.407
**Kurtosis (excess)**	-0.862	1.519	-0.708	0.264	0.226	-0.658	3.727	-0.8	-0.066	3.108	-0.93	0.154	0.576

Table of mean, standard deviation, skewness and excess kurtosis for all variables of interest, including the items from the heuristic strategy questionnaire.
